# Supplementary material for: Combinatorial assembly platform enabling engineering of genetically stable metabolic pathways in cyanobacteria
Source: Nucleic Acids Res. 2021 Sep 23;49(21):e123. doi: 10.1093/nar/gkab791 (PMC8643660; doi:10.1093/nar/gkab791)
Supplement: gkab791_Supplemental_File [file gkab791_supplemental_file.pdf]

Supplementary Information

Combinatorial assembly platform enabling engineering of genetically stable metabolic pathways in cyanobacteria

George M. Taylor[1], Andrew Hitchcock[2] and John T. Heap*[1,3]

[1] Imperial College Centre for Synthetic Biology, Department of Life Sciences, Imperial College London, London, SW7 2AZ, United Kingdom

[2] Department of Molecular Biology and Biotechnology, University of Sheffield, Firth Court, Western Bank, Sheffield, S10 2TN, United Kingdom

[3] School of Life Sciences, The University of Nottingham, University Park, Nottingham, NG7 2RD, United Kingdom

*** Corresponding author**
John T. Heap, email: john.heap@nottingham.ac.uk

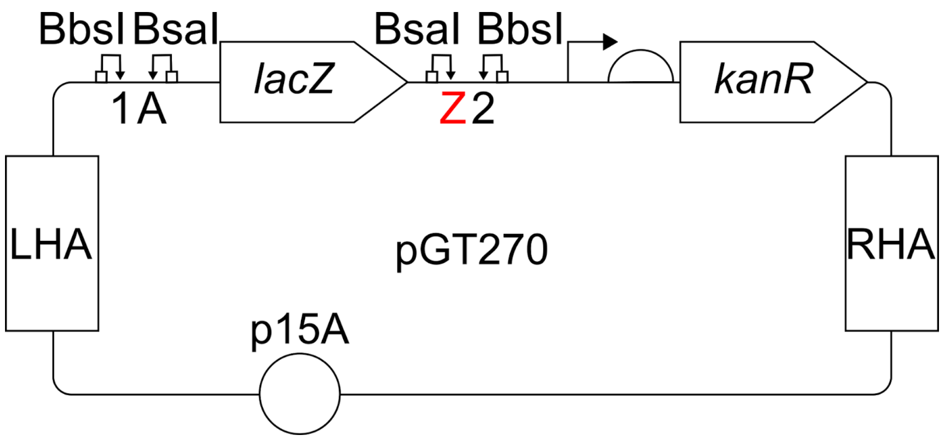

Figure S1. *E. coli-Synechocystis* shuttle vector pGT270 containing Level 2 Start-Stop Assembly cassette. The plasmid contains the low-copy number origin of replication p15A that allows replication of the shuttle vector in *E. coli*. Left homology arm (LHA) and right homology arm (RHA) mediate integration at the *ssl0410* locus of the *Synechocystis* genome. Between the homology arms is a Level 2 Start-Stop Assembly cassette, which includes a *lacZ*α cassette for blue/white screening, two outward-facing BsaI recognition sites with corresponding A and Z acceptor fusion sites for Level 2 assembly of multiple expression units, and two inward-facing BbsI recognition sites with corresponding 1 and 2 donor fusion sites for Level 3 assemblies (see the original Start-Stop Assembly publication for details of these sites[1]). In addition to the Level 2 Start-Stop Assembly cassette, there is a *kanR* gene encoding an aminoglycoside phosphotransferase which confers resistance to kanamycin in both *E. coli* and *Synechocystis*.

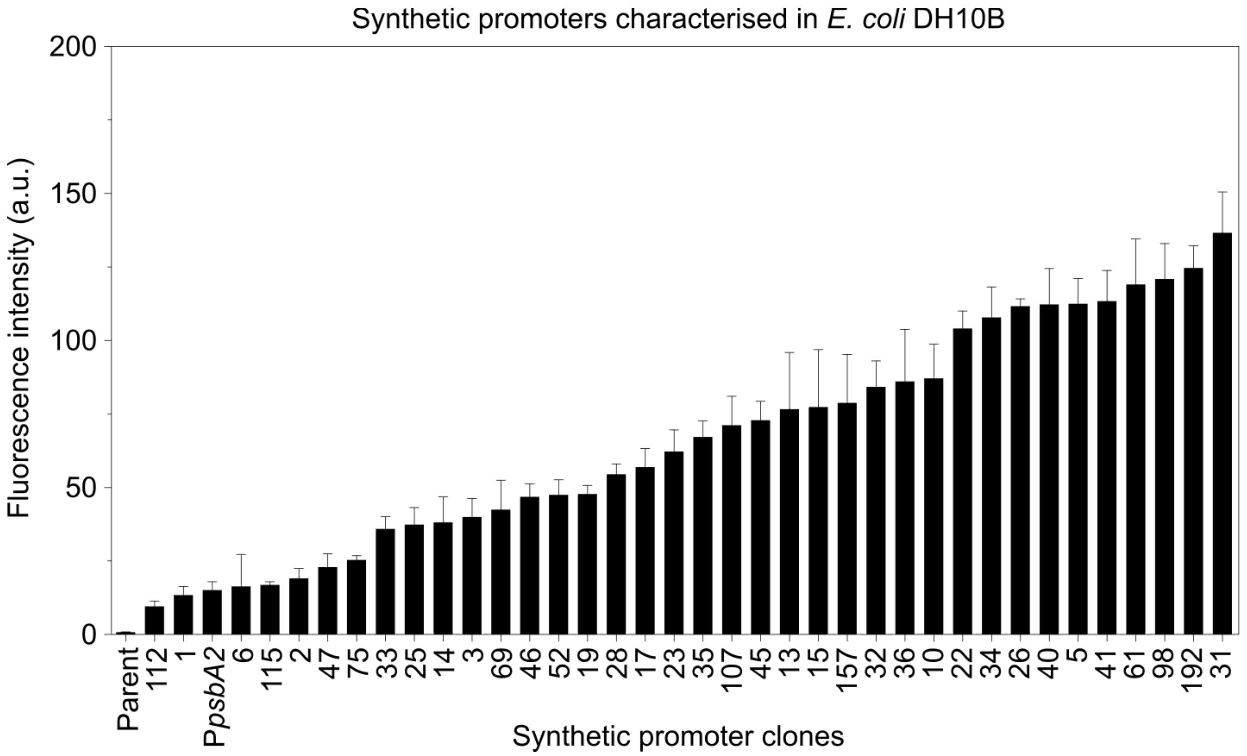

Figure S2. Synthetic promoter library characterisation in *E. coli* DH10B. *E. coli* DH10B cells were transformed with plasmids containing the 37 promoter clones derived from the SPL. Transformant colonies were cultured overnight at 37 °C in LB medium supplemented with kanamycin (50 µg ml^{-1}), subcultured into fresh LB medium, grown for 4 hours, then the fluorescence of 10,000 cells was measured by flow cytometry. The fluorescence intensities presented represent the fluorescence of cells with wild-type background fluorescence subtracted. 'Parent' plasmid pATM2 was used as the template to generate the SPL, and uses the native strong promoter Psll1120 to drive expression of EYFP[2]. 'P*psbA2*' indicates the plasmid pATM10 used as a positive control, in which the strong native promoter P*psbA2* is used to drive expression of EYFP, using the same insulator and synthetic RBS as the SPL design. Error bars represent the standard deviation of five biological replicates.

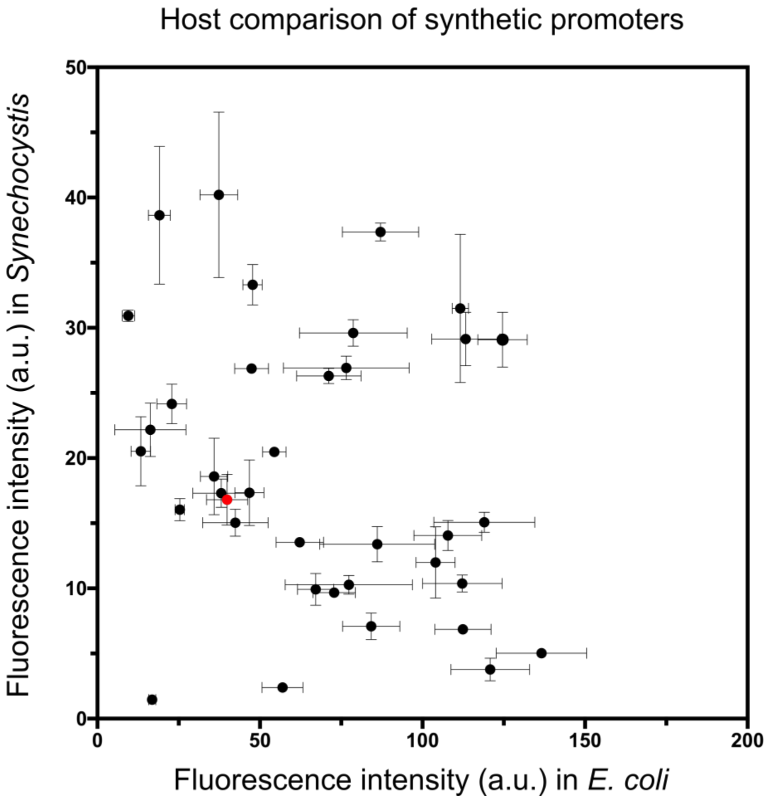

Figure S3. Comparison of synthetic promoters from the SPL in *E. coli* DH10B and *Synechocystis*. EYFP fluorescence intensity generated by each synthetic promoter in *E. coli* (as described in Figure S2) was plotted against the EYFP fluorescence intensity generated by the same synthetic promoter in *Synechocystis* (as described in Figure 1b and Figure S8). No correlation in the performance of the synthetic promoters was observed between *E. coli* and *Synechocystis* (R^2 = 0.05). SPL promoter clone 3 (red dot) is used in the RBS library design. Error bars represent standard deviations of five biological replicates for *E. coli* and three biological replicates for *Synechocystis*.

Construct	Sequence
pATM2	TCGGGTAAGTTTATAATATAC**AAAGGAGGT**AGAA
RBS library design 1	NNNNNNNNNNNNNNN**AAAGGAGGT**NNNNN
RBS library design 2	NNNNNNNNNNNNNNN**NAAGGAGGN**NNNNN
RBS library design 3	NNNNNNNNNNNNNNN**NNAGGAGGN**NNNNN
RBS library design 4	NNNNNNNNNNNNNNN**NNNGGAGGN**NNNNN
RBS library design 5	NNNNNNNNNNNNNNN**NNNNGAGGN**NNNNN
RBS library design 6	NNNNNNNNNNNNNNN**NNNNNAGGN**NNNNN
RBS library design 7	NNNNNNNNNNNNNNN**NNNNNNGGN**NNNNN
RBS library design 8	NNNNNNNNNNNNNNN**NNNNNNNGN**NNNNN
RBS library design 9	NNNNNNNNNNNNNNN**NNNNNNNNN**NNNNN
pGT77	AGAGCAGGATTAGAG**GGAGGT**CAGAGA

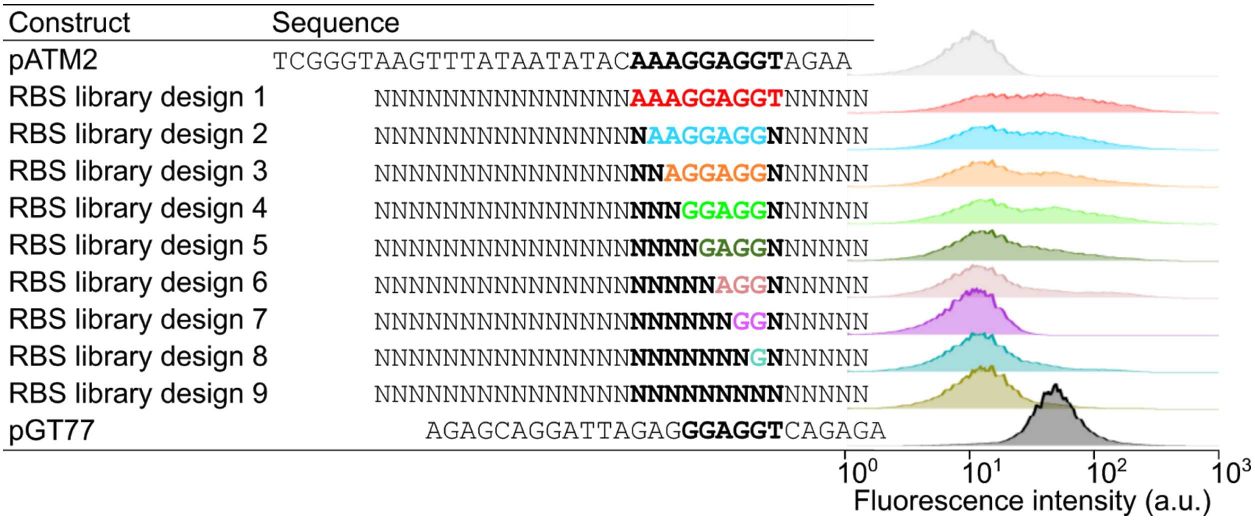

Figure S4. The distribution of RBS strengths in each of the nine RBS library designs in *E. coli*. The sequence for each of the library designs shows the different degrees of randomisation ('N' represents an equal mixture of the four bases A, T, C and G) that was introduced in the nine RBS library designs. Flow cytometry histograms show 10,000 *E. coli* cell counts normalised to the maximum (in order to visualise distribution rather than absolute values) for pATM2 as a negative reference, pGT77 as a positive reference and a pool of several hundred transformants for each of the nine library designs.

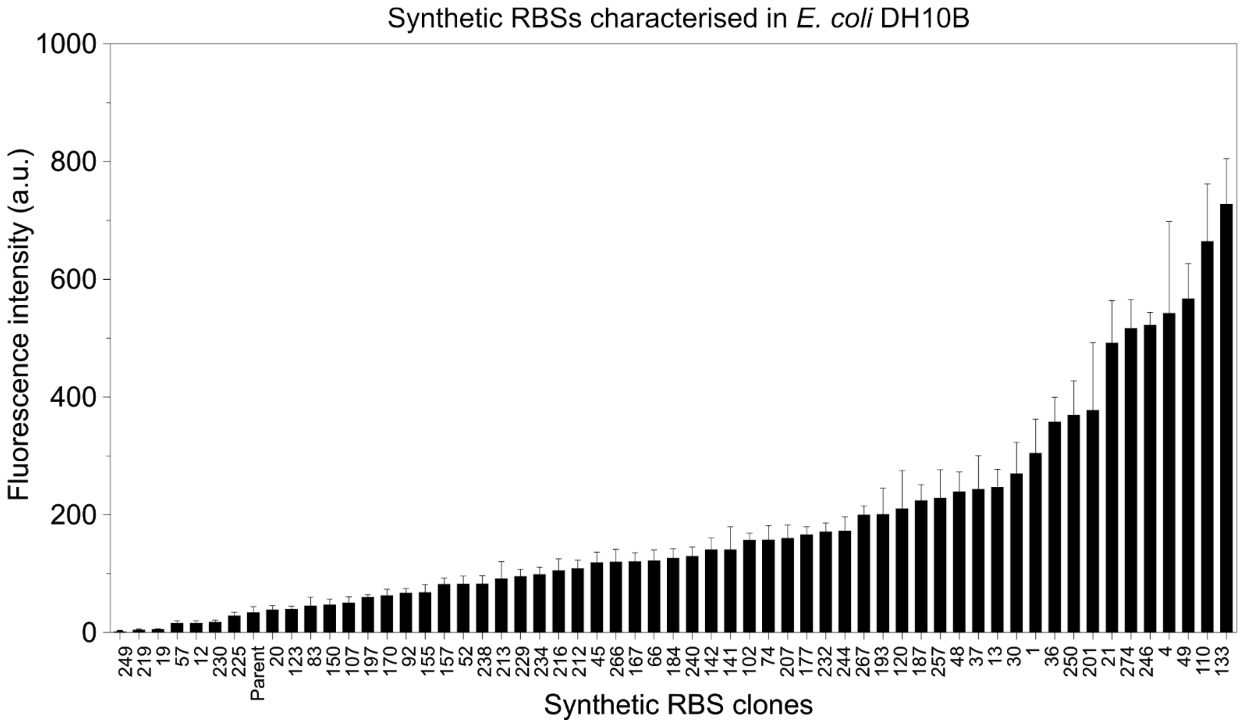

Figure S5. RBS library characterisation in *E. coli* DH10B. *E. coli* DH10B cells were transformed with plasmids containing the 58 RBS clones (under promoter SPL clone 3) derived from the RBS library. Transformant colonies were cultured overnight at 37 °C in LB medium supplemented with kanamycin (50 μg ml^{-1}), subcultured into fresh LB medium, grown for 4 hours, then the fluorescence of 10,000 cells was measured by flow cytometry. The fluorescence intensities presented represent the fluorescence of cells with wild-type background fluorescence subtracted. 'Parent' plasmid pGT77 was used as the template to generate the RBS libraries, and uses the mid-strength promoter SPLc3, which was derived from the SPL. Error bars represent the standard deviation of five biological replicates.

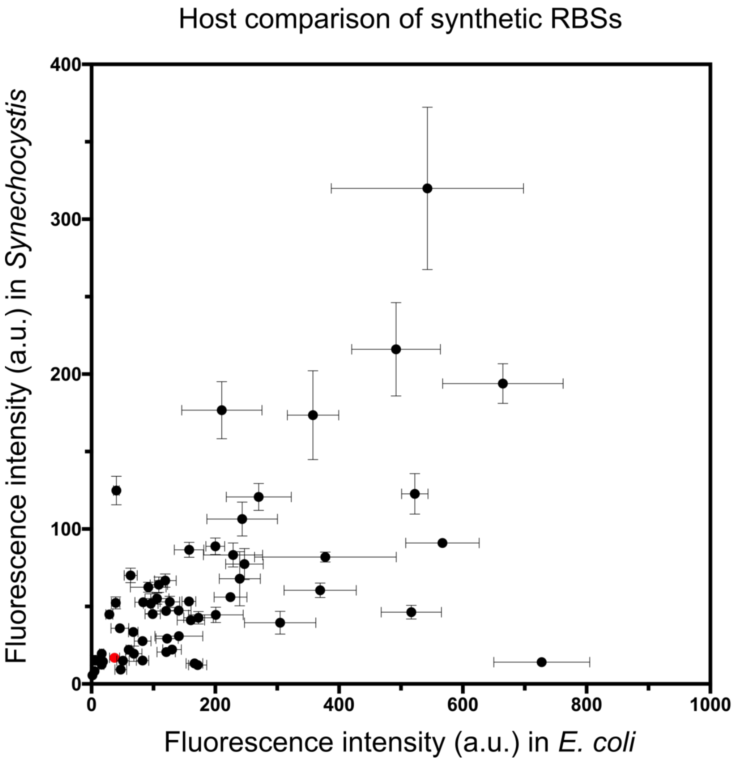

Figure S6. Comparison of RBSs between *E. coli* DH10B and *Synechocystis* sp. PCC 6803. EYFP fluorescence intensity caused by each synthetic RBS in *E. coli* (as described in Figure S5) plotted against the EYFP fluorescence intensity caused by the same RBS in *Synechocystis* (as described in Figure 1c). A weak correlation in the performance of the RBSs was observed between *E. coli* and *Synechocystis* (R^2 = 0.34). pGT77 (SPL promoter clone 3; red dot) was included as the positive reference containing the promoter used for the RBS library design. Error bars represent standard deviations of five biological replicates for *E. coli* and three biological replicates for *Synechocystis*.

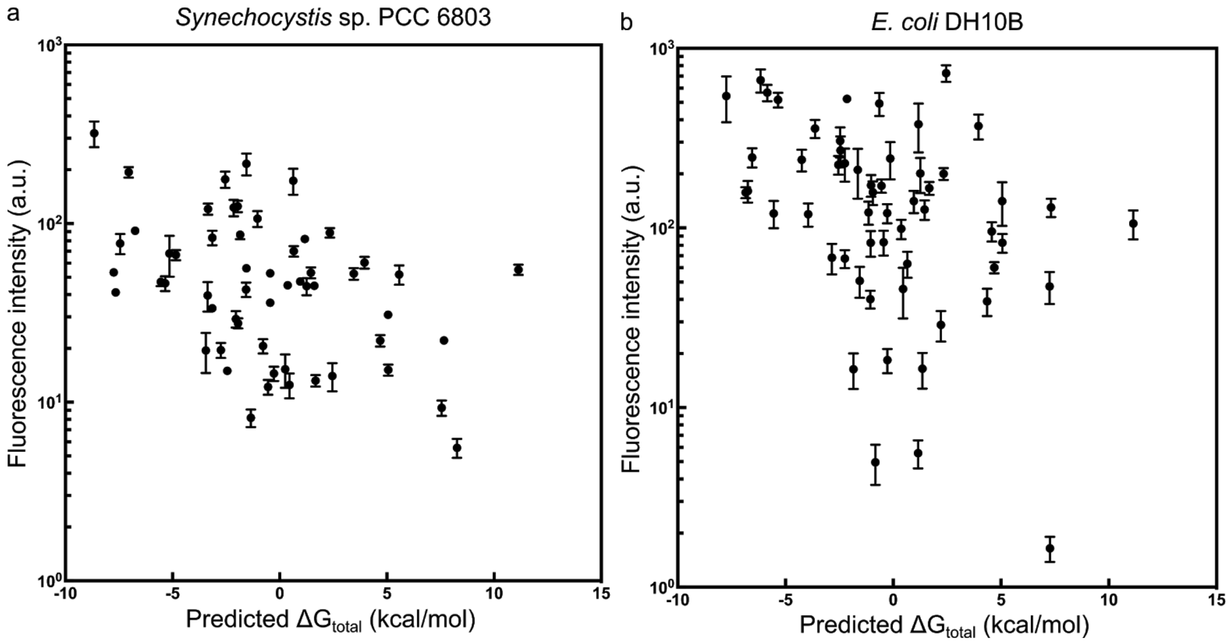

Figure S7. Comparison of the predicted and measured strength of the synthetic RBSs in *Synechocystis* and *E. coli*. The log10 EYFP fluorescence caused by the synthetic RBSs compared to their predicted ΔG_{tot} in (a) *Synechocystis* (R^2 = 0.151) and (b) *E. coli* DH10B (R^2 = 0.149) calculated using the RBS Calculator v2.0[3]. The error bars represent the standard deviation of five biological replicates.

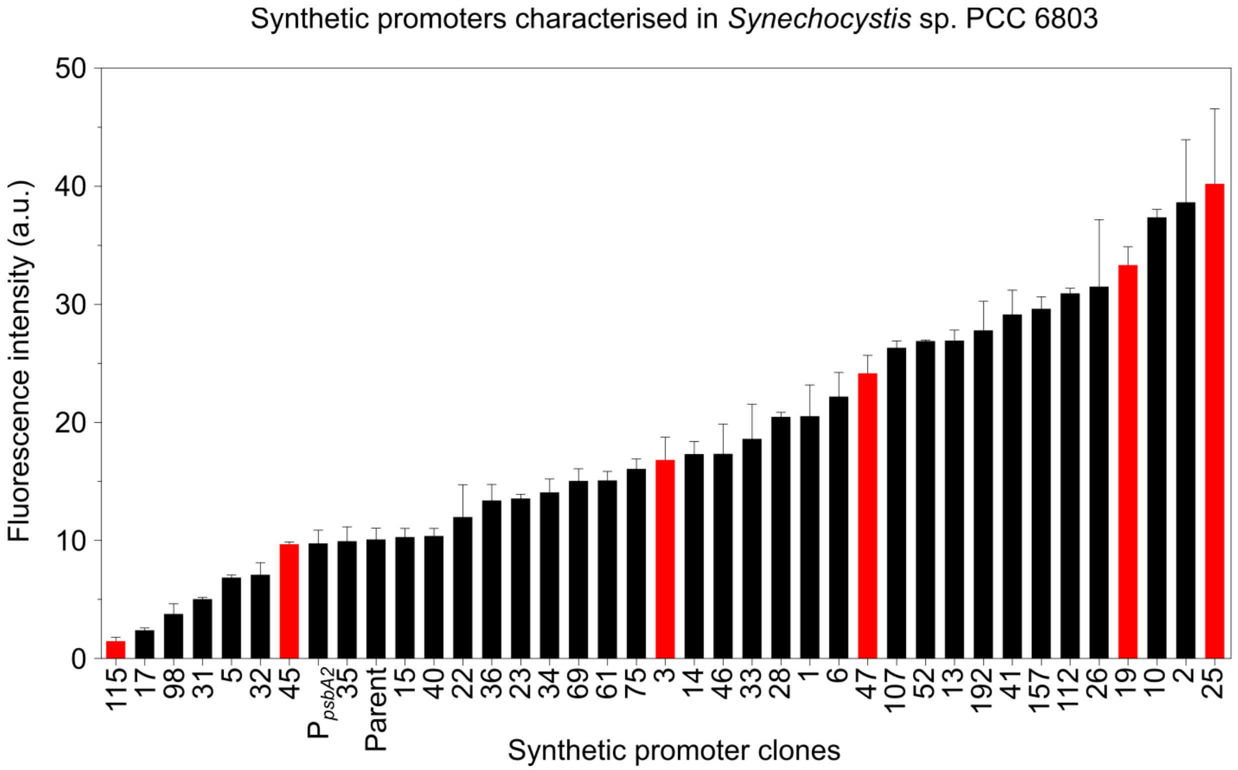

Figure S8. Synthetic promoter library characterisation in *Synechocystis*. *Synechocystis* clones with different synthetic promoters derived from the SPL were cultured in BG11 medium in photoautotrophic conditions with constant light and the fluorescence intensity of 10,000 cells in mid-linear phase of growth was measured using flow cytometry. Fluorescence intensities presented represent the fluorescence of cells with background fluorescence subtracted. 'Parent' represents a transformant of the plasmid pATM2, from which the synthetic promoter library was derived. 'P*psbA2*' indicates the plasmid pATM10 used as a positive control, the plasmid uses the strong native promoter P*psbA2* to drive expression of EYFP, using the same insulator and synthetic RBS as the SPL design. The red bars represent the subset of six chosen promoters. Error bars represent the standard deviation of three biological replicates.

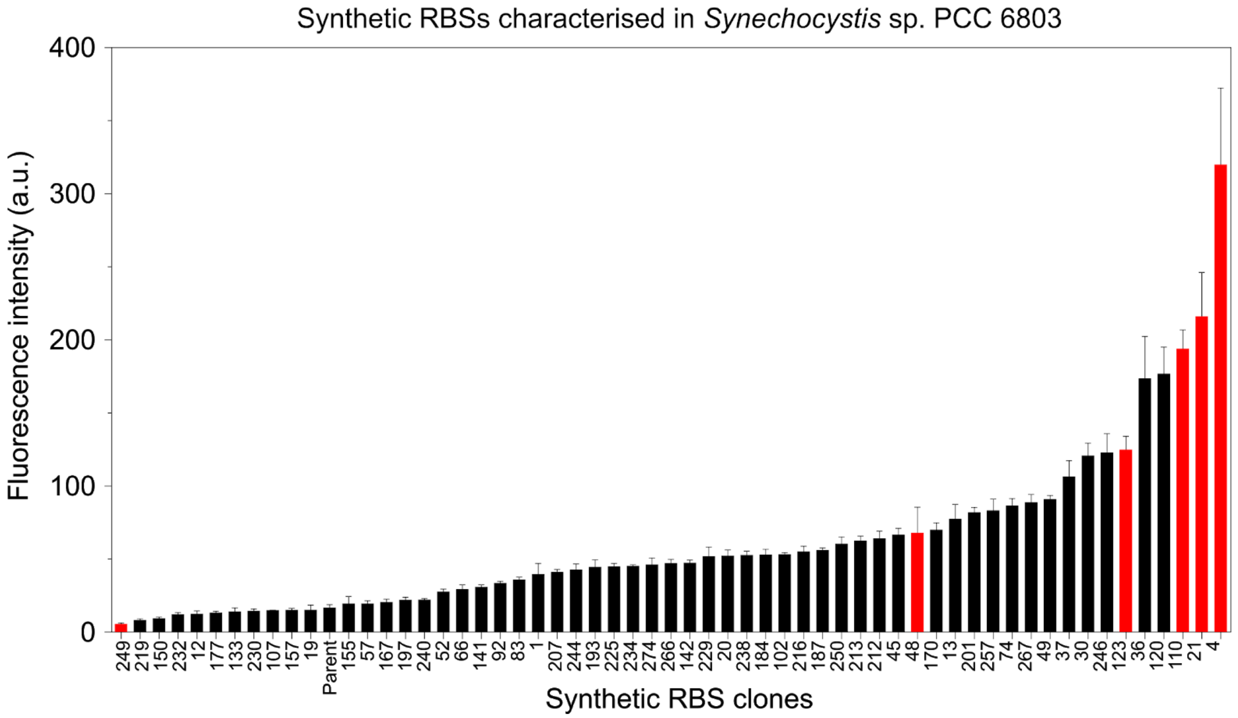

Figure S9. RBS library characterisation in *Synechocystis* sp. PCC 6803. *Synechocystis* clones with different synthetic RBSs derived from the RBS library were cultured in BG11 medium in photoautotrophic conditions with constant light and the fluorescence intensity of 10,000 cells in mid-linear phase of growth was measured using flow cytometry. Fluorescence intensities presented represent the fluorescence of cells with background fluorescence subtracted. ‘Parent’ indicates a transformant of the plasmid pGT77, from which the RBS library was derived. The red bars represent the subset of six chosen RBSs. Error bars represent the standard deviation of three biological replicates.

Design and assembly of rational pathway designs (pGT432, pGT433, pGT434 and pGT435)

Design

High expression design pGT432	SPLc19	RBSc21
Medium expression design pGT433	SPLc3	RBSc123
Low burden design pGT434	SPLc19	RBSc48
High burden design pGT435	SPLc45	RBSc21

Assembly

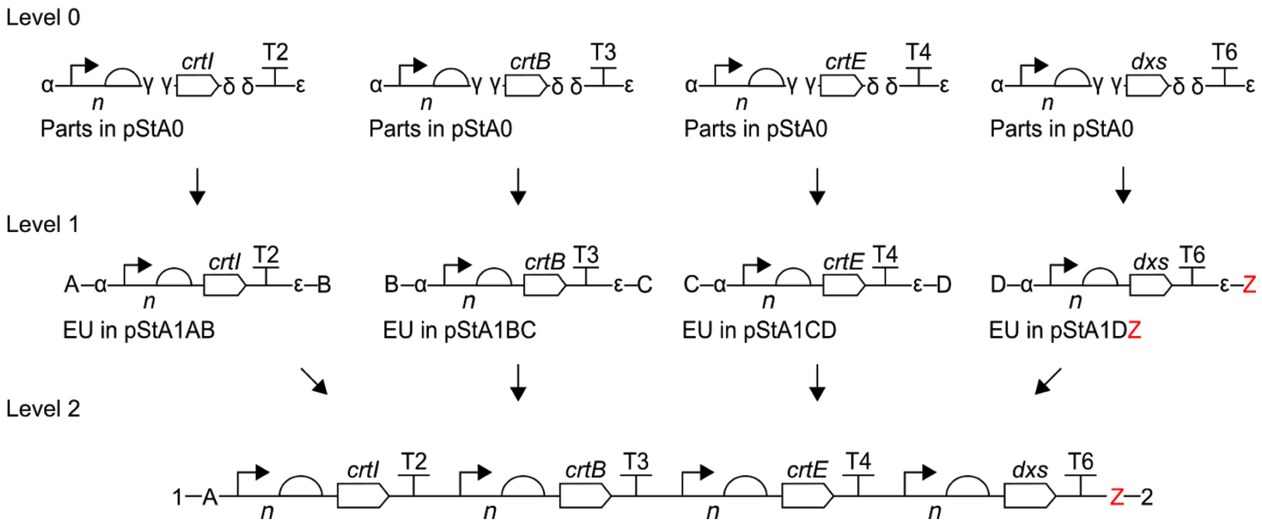

Figure S10. Design and assembly of four individual pathway-encoding constructs. Four pathway design strategies were used: high expression (pGT432), medium expression (pGT433), low burden (pGT434) and high burden (pGT435). Each construct was individually assembled using Start-Stop Assembly in the destination vector pGT270. pGT434 was not cloneable in *E. coli* in three independent attempts. '*n*' shows the uncertain representation of each promoter and RBS at each position following assembly. T2 = terminator L3S2P21, T3 = terminator ECK120033737, T4 = terminator ECK120019600 and T6 = ECK120015170. Assembled insert size = 5,818 bp. Total size of plasmids including assembled insert = 10,164 bp.

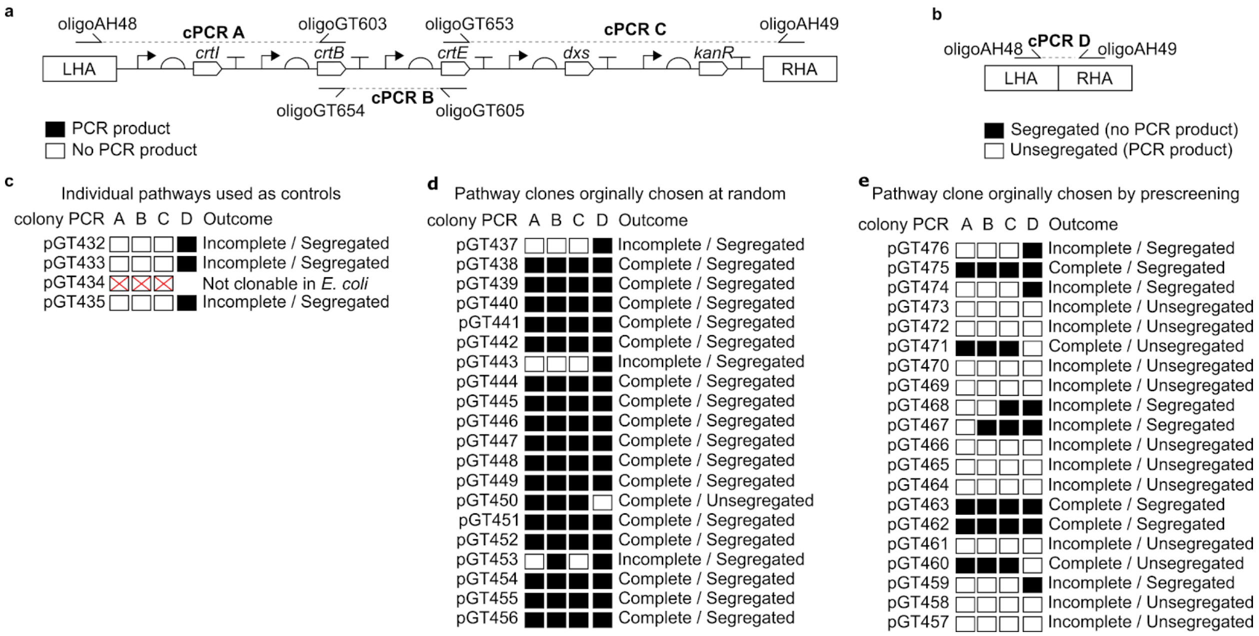

Figure S11. PCR screening for genetic stability of pathway constructs in *Synechocystis*. (a) To confirm whether each pathway construct was complete or incomplete, three separate colony PCR reactions (A, B and C) were used, because complete pathways were too large to amplify as one fragment. (b) To assess segregation, colony PCR D was used, which tested for the presence of a wild-type copy of the integration locus. The presence of a wild-type copy of the locus indicates that the introduced pathway had not fully segregated. The absence of the wild-type amplicon suggests that the pathway construct was present in every copy of the chromosome. Complete pathways were too large to amplify therefore this reaction only screened for the presence or absence of the wild-type locus. (c) The outcome of the four colony PCR reactions for the four rational pathway designs. (d) The outcome of the four colony PCR reactions for the 20 pathway variants originally chosen at random. (e) The outcome of the four colony PCR reactions for the 20 pathway variants originally chosen by prescreening in *E. coli*. Black boxes represent the desired outcome, white boxes represent the unwanted outcome. In the case of PCR reactions A, B and C, black boxes represent the presence of that pathway amplicon, whereas white boxes represent its absence. In the case of PCR reaction D, a black box represents a segregated pathway construct, whereas a white box represents an unsegregated pathway construct.

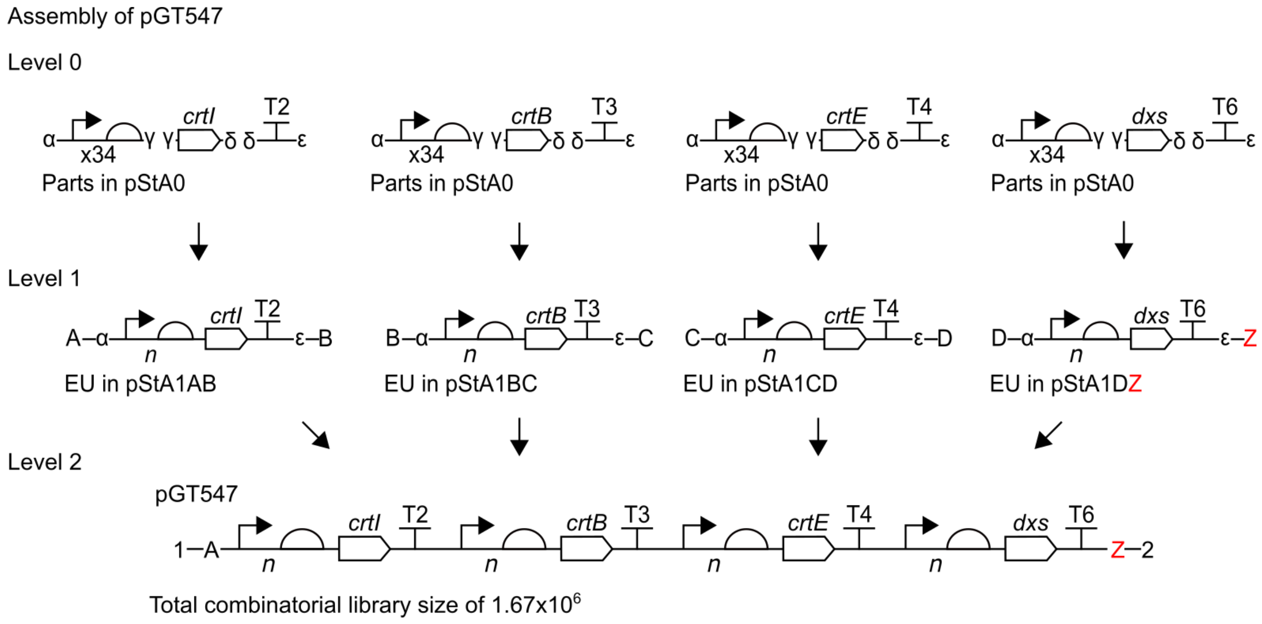

Figure S12. Combinatorial assembly of lycopene pathway library pGT547. A library of lycopene pathway construct variants was assembled as shown using Start-Stop Assembly. The destination vector was pGT270. An equimolar mixture of the 34 promoter-RBS composite parts labelled as 'x34' was used in Level 1 assemblies. '*n*' shows the uncertain representation of each promoter and RBS at each position following assembly. The maximum library size was 34^4 = 1.3×10^6. T2 = terminator L3S2P21, T3 = terminator ECK120033737, T4 = terminator ECK120019600 and T6 = ECK120015170. Assembled insert size = 5818 bp. Total size of plasmids including assembled insert = 10,164 bp.

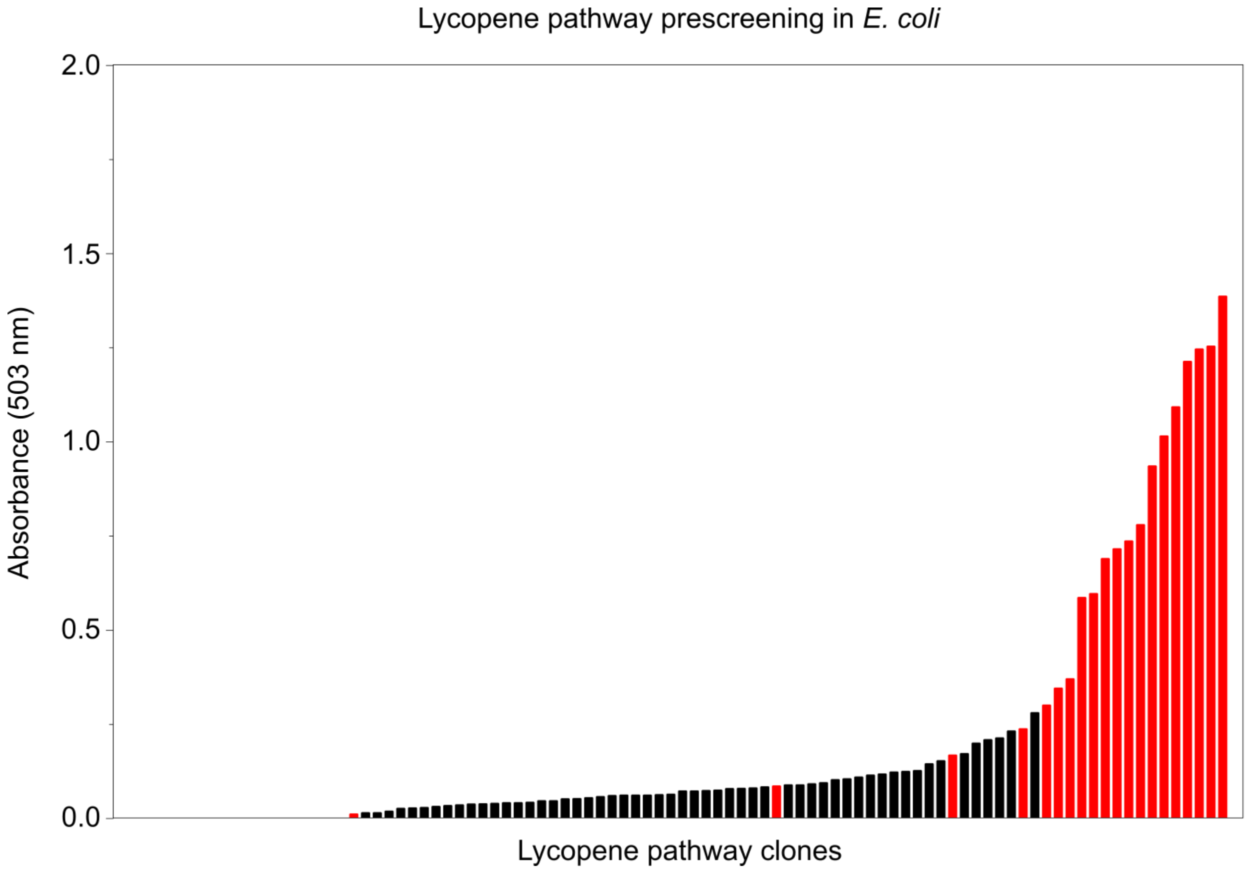

Figure S13. Prescreening of construct variants for lycopene production in *E. coli*. As described in the main text, 96 *E. coli* transformant clones were picked at random from the combinatorial lycopene overproduction pathway library and screened for lycopene production after 48 h of growth by spectrophotometry (as described in Methods). The absorbance at 503 nm of each clone is presented with background absorbance subtracted. Of these 96 clones, twenty (shown in red) were chosen for further study, of which ten were the strongest lycopene producers and the other ten were evenly-distributed throughout the observed range of lycopene concentrations. Order of pathway clones from least to most lycopene produced, left to right: clone 34, 67, 93, 10, 70, 86, 5, 14, 46, 51, 74, 89, 37, 22, 41, 49, 61, 91, 78, 94, 58, 44, 82, 39, 64, 54, 50, 27, 15, 53, 19, 32, 38, 56, 59, 33, 43, 85, 52, 3, 88, 30, 28, 25, 47, 48, 57, 62, 9, 83, 8, 11, 68, 18, 80, 26, 2, 31, 65, 71, 84, 40, 36, 96, 6, 73, 55, 60, 1, 81, 66, 63, 35, 72, 13, 42, 16, 23, 12, 45, 7, 21, 79, 87, 17, 75, 4, 24, 90, 95, 77, 92, 20, 69, 76.

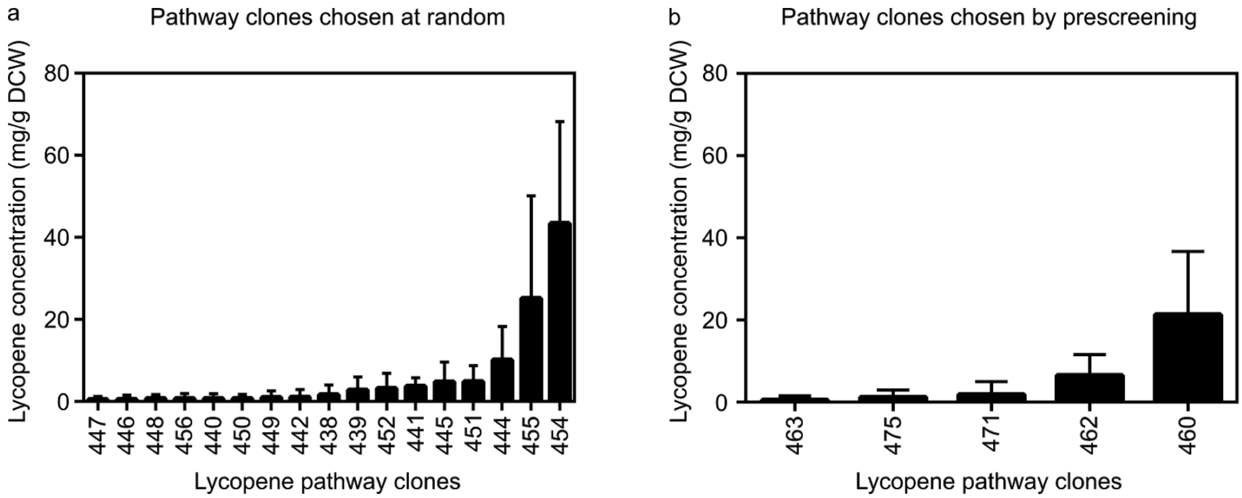

Figure S14. Lycopene concentrations in *E. coli* produced by pathway construct variants which were stable in *Synechocystis*. *E. coli* DH10B cells were transformed with the lycopene pathway construct variants chosen either (a) at random or (b) after prescreening in *E. coli*. Only construct variants that were genetically stable in *Synechocystis* are shown. Transformant clones were grown for 24 hours at 30 °C in LB medium supplemented with kanamycin (50 µg ml^{-1}), subcultured into fresh LB medium, grown for 48 hours, then lycopene concentration was determined. The error bars represent the standard deviation of three independent biological replicates. Variant names are shown as numbers only, omitting the initial ‘pGT’.

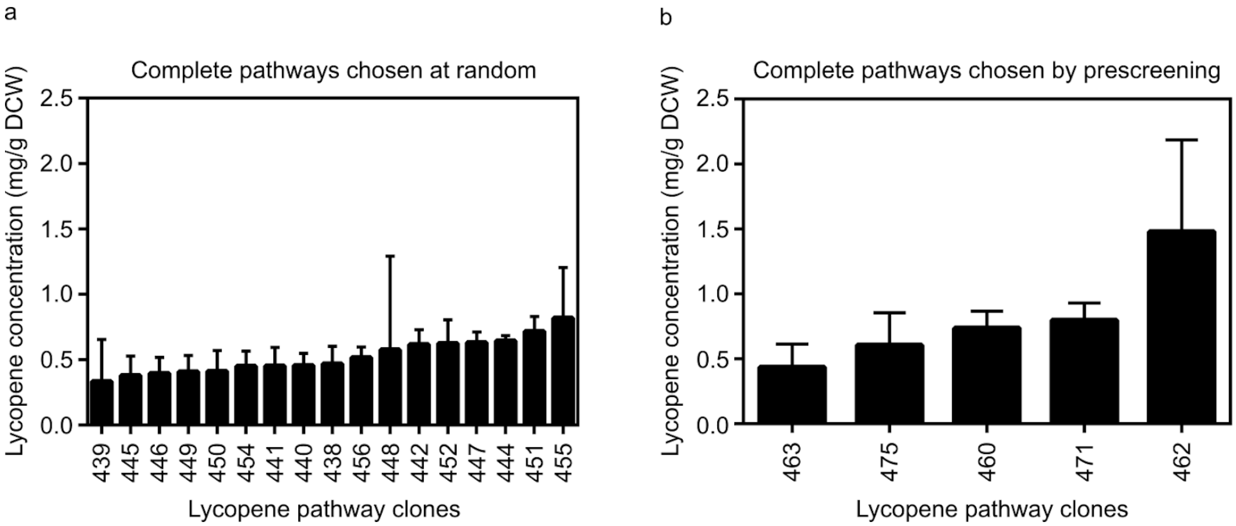

Figure S15. Lycopene concentrations in *Synechocystis* produced by pathway construct variants, grouped by strategy originally used to identify variants. *Synechocystis* cells containing complete pathway constructs (a) chosen at random in *E. coli* or (b) chosen by prescreening in *E. coli* were grown in BG11 medium supplemented with kanamycin (30 µg ml^{-1}), in photoautotrophic conditions and constant light for 2 days, subcultured into fresh BG11 medium and grown for two weeks prior to determination of lycopene concentration. The error bars represent the standard deviation of three independent biological replicates. Variant names are shown as numbers only, omitting the initial 'pGT'.

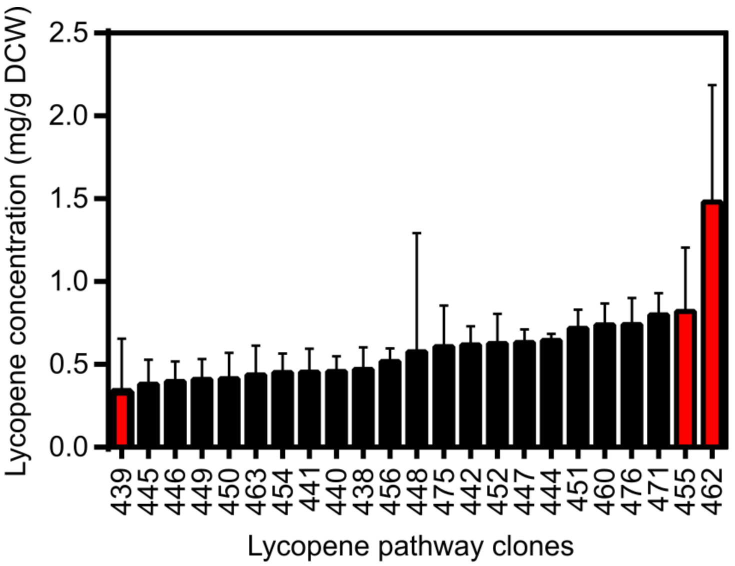

Figure S16. Lycopene concentrations in *Synechocystis* produced by pathway construct variants, showing three variants chosen for further study. Lycopene concentrations of the 17 pathway variants chosen at random (Figure 4) and the six pathway variants chosen by prescreening in *E. coli* (Figure 4) characterised in *Synechocystis*. Three variants chosen for further characterisation are shown in red: a low producing strain pGT439, a medium producing strain pGT455, and a high producing strain pGT462. The error bars represent the standard deviation of three independent biological replicates. Variant names are shown as numbers only, omitting the initial 'pGT'.

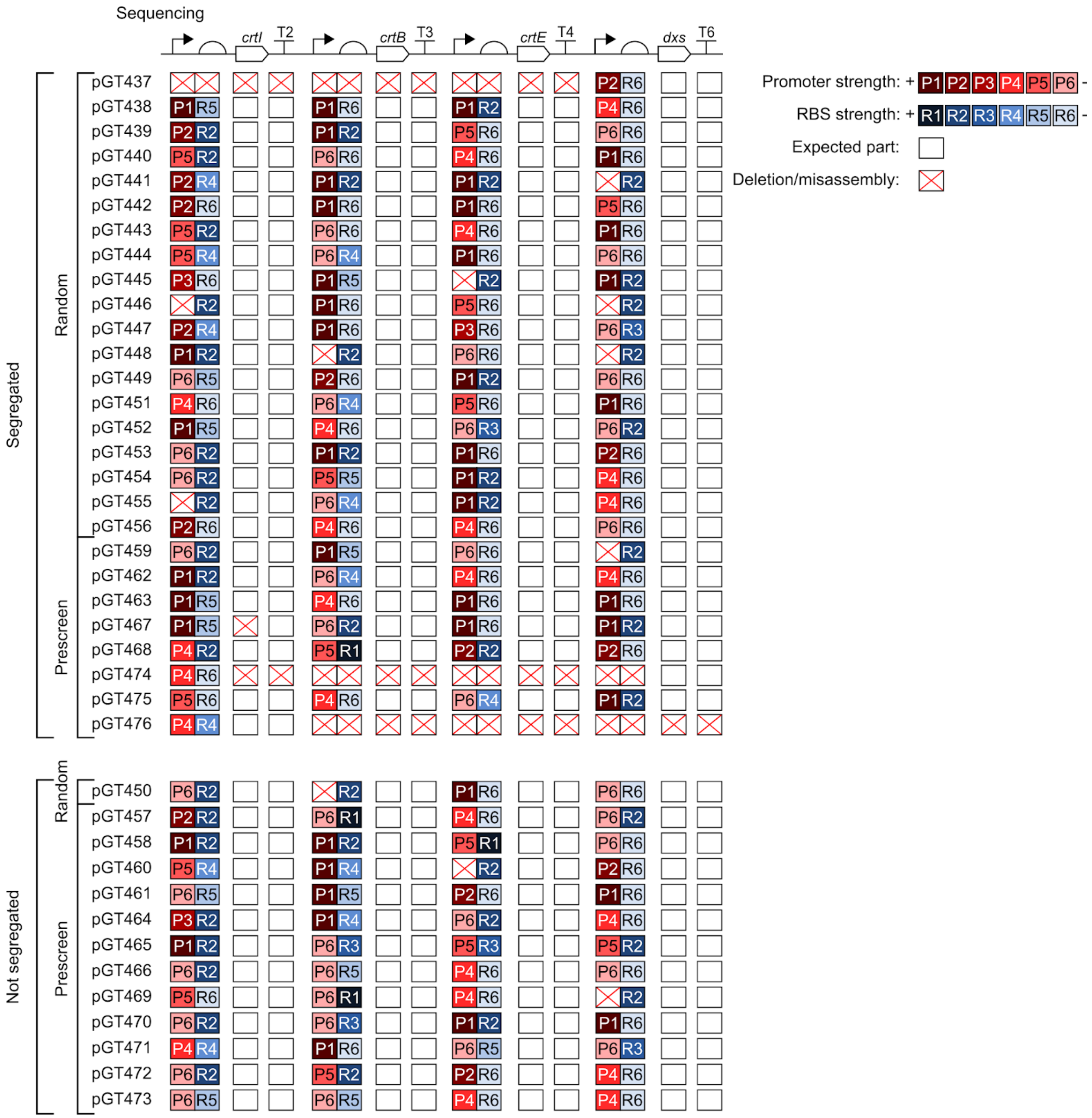

Figure S17. Sequencing results of lycopene pathway variants grouped first by pathway segregation, then by strategy used to identify variants. All 40 pathway variants (20 pathway variants chosen at random and 20 pathway variants chosen by prescreening) were sequenced. Promoters, in descending order of strength as characterised using EYFP: P1 = SPLc25, P2 = SPLc19, P3 = SPLc47, P4 = SPLc3, P5 = SPLc45 and P6 = SPLc17. RBSs, in descending order of strength as characterised using EYFP: R1 = RBSc4, R2 = RBSc21, R3 = RBSc110, R4 = RBSc123, R5 = RBSc48 and R6 = RBSc249. In the sequencing results the white box indicates presence of the expected part and the red cross represents a deletion or misassembly.

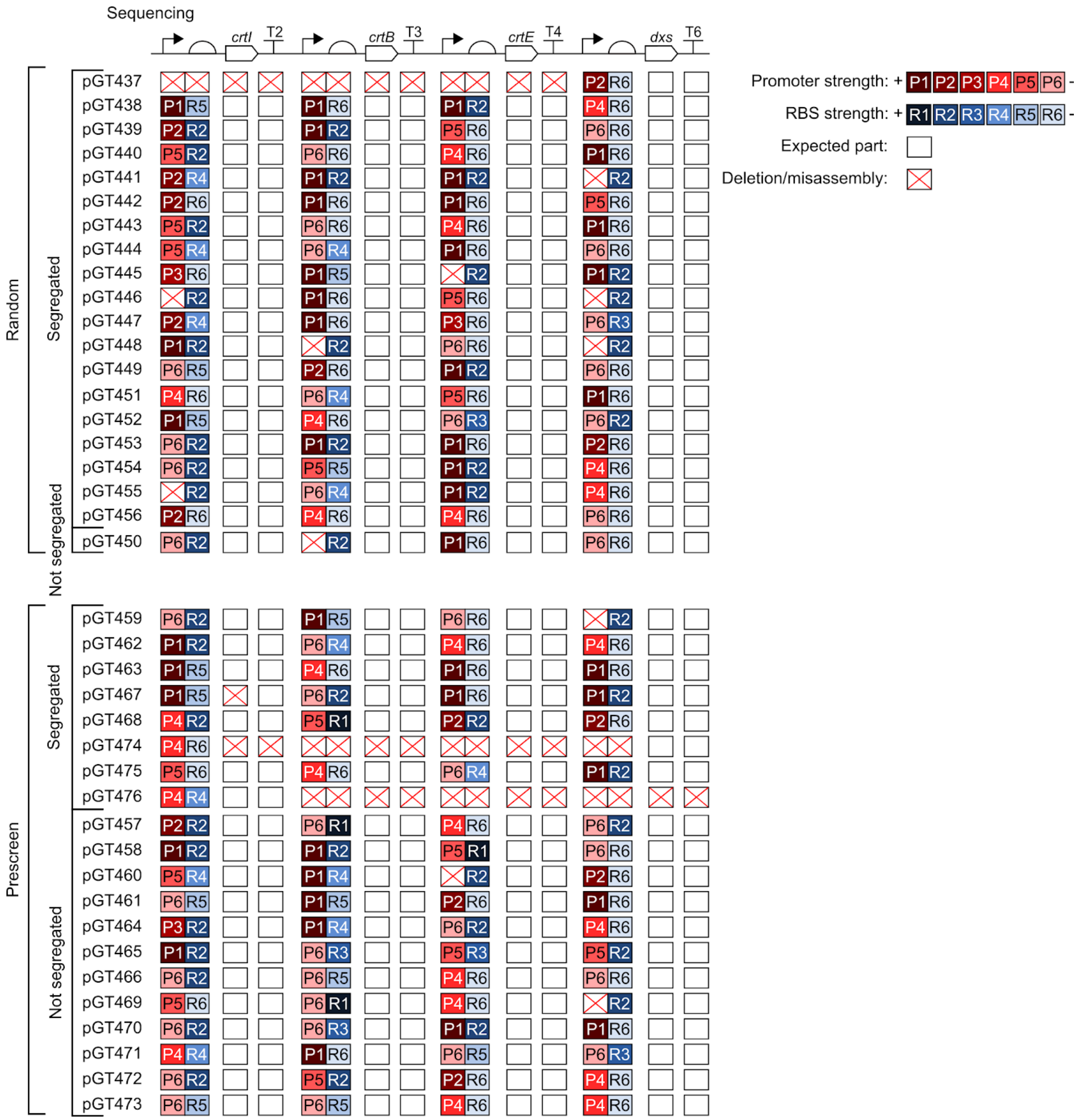

Figure S18. Sequencing results of lycopene pathway variants grouped first by strategy used to identify variants, then by pathway segregation. All 40 pathway variants (20 pathway variants chosen at random and 20 pathway variants chosen by prescreening) were sequenced. Promoters, in descending order of strength as characterised using EYFP: P1 = SPLc25, P2 = SPLc19, P3 = SPLc47, P4 = SPLc3, P5 = SPLc45 and P6 = SPLc17. RBSs, in descending order of strength as characterised using EYFP: R1 = RBSc4, R2 = RBSc21, R3 = RBSc110, R4 = RBSc123, R5 = RBSc48 and R6 = RBSc249. In the sequencing results the white box indicates presence of the expected part and the red cross represents a deletion or misassembly.

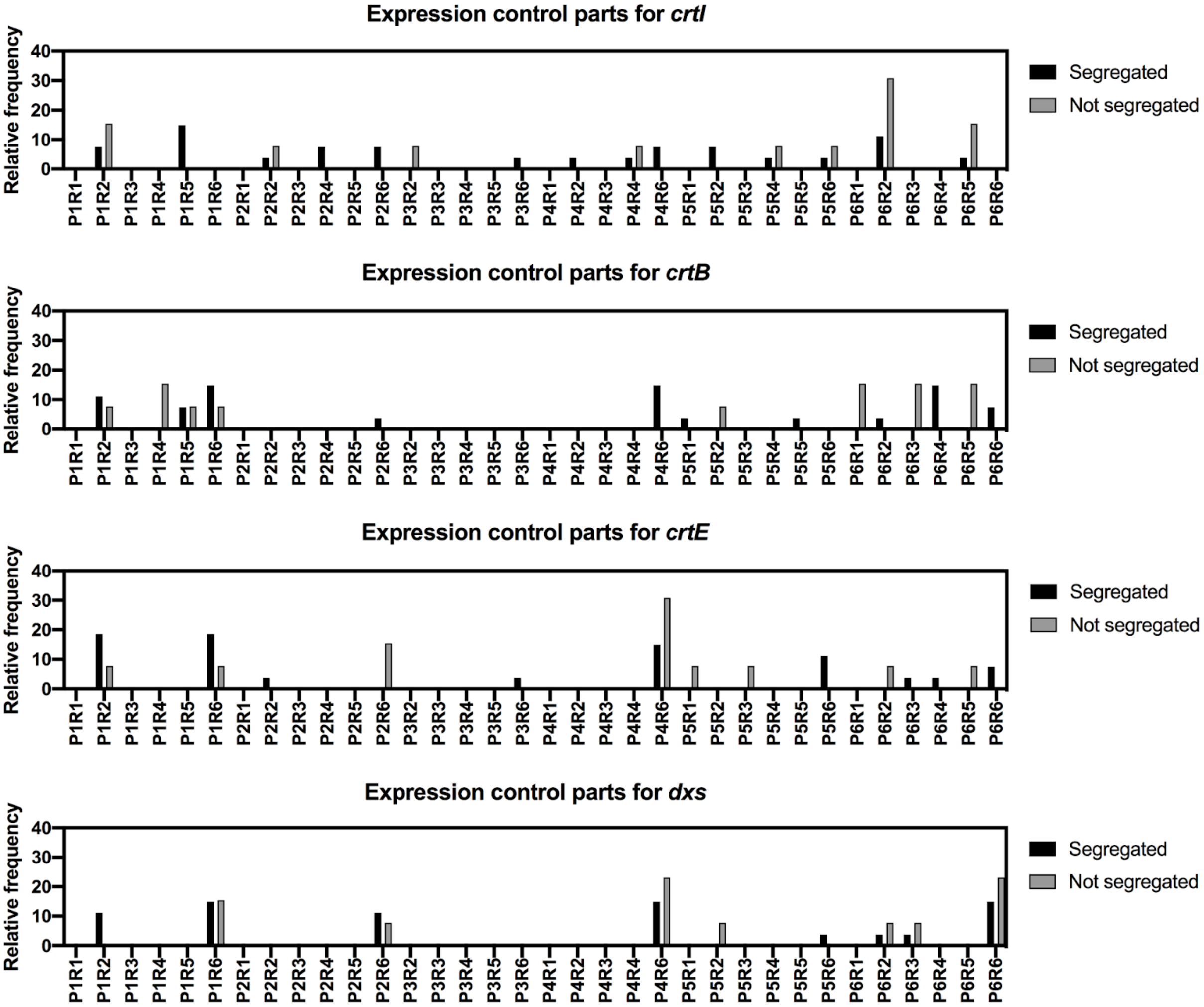

Figure S19. Frequencies of promoter and RBS combinations in pathway variants. The relative frequencies of each promoter and RBS combination found in segregated and unsegregated pathway variants. Segregated pathway variants are displayed as black bars. Non-segregated pathway variants are displayed as grey bars. Promoters, in descending order of strength as characterised using EYFP: P1 = SPLc25, P2 = SPLc19, P3 = SPLc47, P4 = SPLc3, P5 = SPLc45 and P6 = SPLc17. RBSs, in descending order of strength as characterised using EYFP: R1 = RBSc4, R2 = RBSc21, R3 = RBSc110, R4 = RBSc123, R5 = RBSc48 and R6 = RBSc249.

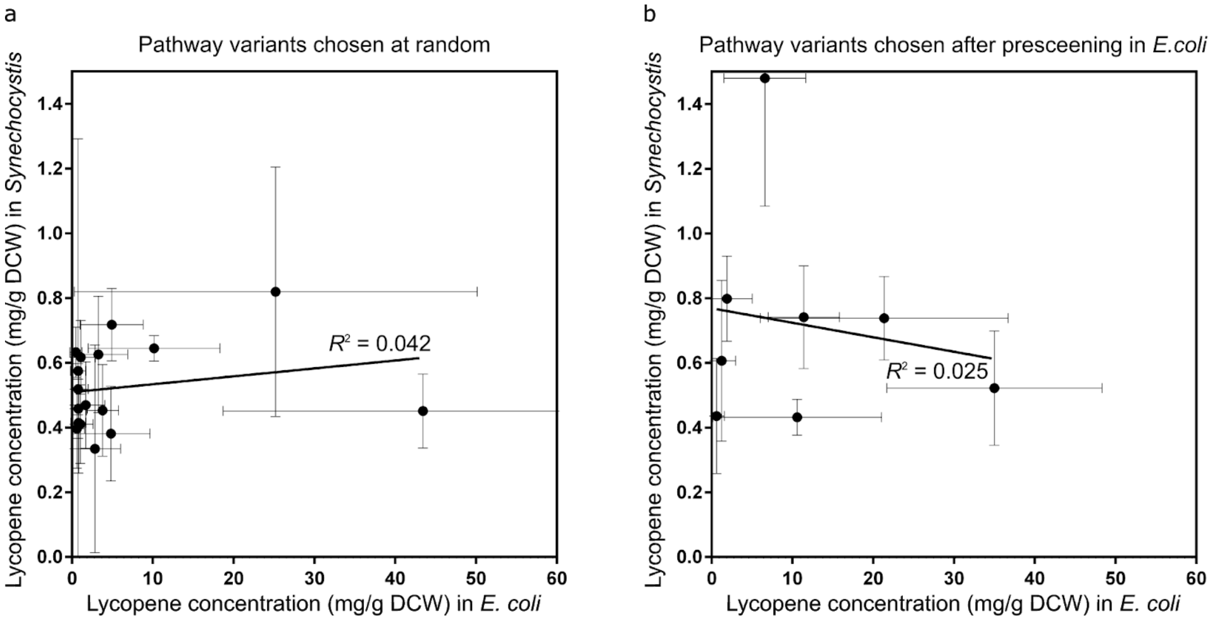

Figure S20. Comparison of lycopene concentrations produced by pathway construct variants in *E. coli* and *Synechocystis*. (a) Lycopene concentrations in *E. coli* (Figure S14a) compared against those in *Synechocystis* (Figure S17a) for the pathway variants chosen at random. (b) Lycopene concentrations in *E. coli* (Figure S14b) compared against those in *Synechocystis* (Figure S17b) for the pathway variants chosen by prescreening in *E. coli*. Error bars represent standard deviations of three independent biological replicates for *E. coli* and *Synechocystis*.

Table S1. Sequences of synthetic promoters from the synthetic promoter library. -35 and -10 regions are shown in **bold**.

Synthetic promoter	Sequence (5' - 3')<br>-35 region -10 region
1	CATCGCCCGA**TTGACA**CCTCGCGCCACGCCTGA**TATAAT**GGGGGG
2	TCAAGGGAAG**TTGACA**CGACACCGCTAATTTAT**TATAAT**GAAGAG
3	CCAGATGACC**TTGACA**AGTAACTGCCCCAACAG**TATAAT**ATACCT
5	CAGTGTGGCG**TTGACA**TTCAGTCATGCACCCCG**TATAAT**TGGCGA
6	GCGGGGTTGC**TTGACA**GGATTTACTCGGCAGGG**TATAAT**GGGTAC
10	CGCAGCGGGC**TTGACA**CATCGGCCCAAACTACA**TATAAT**GTGTAT
13	CCCGCAGCTT**TTGACA**CACCGCCACTGACAGTC**TATAAT**TTGGAT
14	TAGTATAAGA**TTGACA**TAATGTAGTCCGTGGAG**TATAAT**GTAATT
15	CACCAGAGGA**TTGACA**GTCCTAGCAGACACGTG**TATAAT**AGTCCC
17	CCCCTAAGCT**TTGACA**CGCAGAGAACACAACCC**TATAAT**GATTCG
19	GCGGCTCGTA**TTGACA**ATTTAACTAAATATCAG**TATAAT**TTAGTT
22	ATAGGCACCG**TTGACA**TACTAGCCAGAAGAAAA**TATAAT**TGGGGG
23	CGGGTGGCAA**TTGACA**TGGCACGAGTCCGTCTA**TATAAT**AACAGA
25	CCACGAAAAA**TTGACA**AGCACTCTGCGCTCTGA**TATAAT**TAAGGT
26	CCCCCACCCC**TTGACA**GCCCGTGGCTCGATGCA**TATAAT**GAATGT
28	CCCACATAAC**TTGACA**GAAGTCCGCAGCGACTT**TATAAT**ACGGGG
31	TTTTATTATA**TTGACA**TCTTTCATACACCTGCA**TATAAT**TCGAGT
32	CGGTCGGCCT**TTGACA**GCGGTTGATTAAGAGAC**TATAAT**GTCTAT
33	CTCGCGGCAA**TTGACA**AAATTGGATCTCGATAT**TATAAT**GGGGGT
34	CCGACATCCT**TTGACA**CGAACTCGCTGGCCCGG**TATAAT**TCTTTA
35	ACACGCCGCC**TTGACA**TCACTTTACGGCACCCA**TATAAT**GGGGGG
36	GCCCCCCGCG**TTGACA**CCCGCCCCGTACTCGCC**TATAAT**TAAGGG
40	CCAAATTATA**TTGACA**TTACCGGGGTCGTGAAC**TATAAT**TCTTTC
41	TGCCCCCCCC**TTGACA**ACCGCTGTCCCGCATCC**TATAAT**TGTTCA
45	CTCGCCACCT**TTGACA**CAGCGGCCCGCTAACTA**TATAAT**AAACGT
46	AGCGCGGACC**TTGACA**CTCCTAGTGACTTACAC**TATAAT**AGCTGG
47	GTGACGTCTA**TTGACA**GCCAGGAGTTACCGAGA**TATAAT**GGAATA
52	CCCCCTCCCC**TTGACA**CCCTCCCATCCCGATGT**TATAAT**GTGTTT
61	TCTAGCCAGA**TTGACA**CTGACATTAAGTACACT**TATAAT**CGGAGG
69	GTCGCCCCCA**TTGACA**AATGTAAGTCGCGGAGG**TATAAT**AGGTGC
75	CTCACGGACC**TTGACA**GACGGGGGGCCCCCAGG**TATAAT**GAGGGG

98	ACCATCTAGT**TTGACA**TCGAAAAGCCAACAACG**TATAAT**CAGCTT
107	CGCGCTACCA**TTGACA**TCCCGTTCCCGCCGCTG**TATAAT**TGATTT
112	CGAAAACACA**TTGACA**CCCTTTTATAATCATGA**TATAAT**TGGATC
115	CCGGACCCCC**TTGACA**CTACGGCCCGCTATGGA**TATAAT**TCACCG
157	CACCGGGCCT**TTGACA**ATACGTATCAAGTACCT**TATAAT**AAGGGG
192	CATTCCGTGC**TTGACA**CATTATTAAACTATGAA**TATAAT**GTGTAA

Table S2. Sequences of synthetic RBSs from the RBS library.

RBS library design	Synthetic RBS	Sequence (5’ - 3’)
RBS library design 1	1	CTCGATTTCGCGAACAAAGGAGGTGAGGT
	4	TATGTCAGTAAAAAAAAAGGAGGTTCGTT
	12	TAAAGTTAAGCGTCCAAAGGAGGTTTAAC
	13	GCGGATAGTGAGGAGAAAGGAGGTGGGGG
	19	ACAAACTCCTCACGGAAAGGAGGTACGGA
	20	GCAAATTCTCGTGGGAAAGGAGGTCGCGG
	21	TACGAAAGATTTACGAAAGGAGGTTGGTT
	30	ATTTCGTATGAAGGTAAAGGAGGTTGGCA
	36	CTCTAGGGTTTTCTAAAGGAGGTTGAAT
	37	AGTTGACCGTAACGCAAAGGAGGTAGAGC
	45	TCGGCGGCGTAAACTAAAGGAGGTGGAAG
	48	AGTGTGTTCCAAGAGAAAGGAGGTATCTG
	49	AATAAAAGCCAGTTTAAAGGAGGTCTTTG
	52	ATTAGACGACGATCCAAAGGAGGTCCCAG
	57	TGGACTCCTACGAGGAAAGGAGGTGGTA
	66	CTTATGGGTGTTTGAAAGGAGGTGGACG
	74	AGTACTAACGGGTTTAAAGGAGGTCGCGG
	83	GCGGATAGTGAGGAGAAAGGAGGTGGGGG
	92	AAACCGAACAGGCCAAAGGAGGTCAGCT
	102	TTTAGTATTGCTACTAAAGGAGGTTGCAC
	107	TTGGGGCGATTTGGGAAAGGAGGTGCGGG
	110	TAGGTATATGCAATCAAAGGAGGTATATG
	120	ATACTGCATCAATGAAAGGAGGTAGGGG
	123	TGTGTAGGTCCCACCAAAGGAGGTCTGTA
RBS library design 4	133	TTACTATAATAACGTTTCGGAGGGAATAT
	141	TTCAAATAAGCATTGATCGGAGGGTTTAC
	142	GGGATATAACGAAGAGGCGGAGGAGTGCG
	150	TGTTAAGAGGGGGACTGTGAGGGCTGAG
	155	TTACCGGTCAAGGCAAGAGGAGGCTAGTC
	157	TATTATGTGATAATTAGAGGCGTGCA
	167	GTATATGGTGGAGGAGGTAGCGT
	170	CTGATTTAGGGGCCATTTTGAGGAATATG
	177	GTAGGTTGGATATCTGGAGGTGAAGC

RBS library design 4 continued	184	CTGCGTGACAACAGATGTCGAGGTGAAAG
	187	CTCTATTGTGTACTATGAGGAGGCAGCGT
	193	GTTGCGTAACAATAGACGGAGGATGCTT
	197	CTGGTATGGTTCAACGAGGATCGTT
	201	CTATCGGTTATGTGCTAGCGAGGTTTAAT
	207	GTGCAGACGATGAGAAGGGAGGAAAAGA
	212	TTTCGAGGAGGATAGCG
	213	TTTCGAGGAGGATAGCG
	216	TTTCGAGGAGGATAGCG
	219	GAGGGTATAGTGCGTAACGGAGGGGTACG
	225	GTAACGGGGAGTTTAAGTAGAGGGGGTGT
	229	CGGTGTCTTTATTCTAATTGAGGGGGCTT
	230	TTGAAAGGGACCCCACAGTGAGGTAGAGC
	232	TAGGCGTAAGGATCTATCGAGGAAGTGT
	234	TAATGGTCAGCGTGTGCGGAGGAGCGTG
	238	TACGGGTTAGTTTGTTGTGGAGGTTACTT
	240	TCAATTGCTTTGGCGGTGAGAGGCGTGTT
	244	TGGACATTCAAATATTGAAGAGGGGTGTA
	246	CCCTATTAAAAACAGACGGAGGCAGAAT
RBS library design 9	249	GGCGCGAGTCAACGTTCCTGAGGAGTGGC
	250	TCTTTGAGACTTTATTGTGGAGGTTTGGC
	257	CGGAGCAATCAACGAAAGGGGGAAGAGGG
	266	CGGAGCAATCAACGAAAGGGGGAAGAGGG
	267	GTTTCTTAGGTGTTGGAGGTTGGTCTA
	274	AGAGCATTAGGTGATAGGAGGTAAGTA

Table S3. Oligonucleotides used in the study. 5'-phosphorylated oligonucleotides denoted /5Phos/. Fw denotes forward primer. Rv denotes reverse primer.

Oligonucleotide	Sequence (5' – 3')	Comments
oligoATM3	/5phos/NNNNNNNNNNNNNNNNNTGTCAANNNNNNNNNNCTGCAGTCTCCTTTAAATCGG	Rv primer for generating SPL library in pATM2
oligoATM5	/5phos/TATAATNNNNNNCCGGCTTCGGCGGTAAGGTAAGAGCAGGATTAGAGGGAGGTCAGAGAATGGTGAGCAAGGGCGAGG	Fw primer for generating SPL library in pATM2
oligoATM51	/5phos/AGGTATATTATACTGTTGGGGCAG	Rv primer for generating RBS library in pATM2
oligoATM52	NNNNNNNNNNNNNNNAAAGGAGGTNNNNNATGGTGAGCAAGGGCGAGG	Fw primer for generating RBS library design 1 in pATM2
oligoATM53	NNNNNNNNNNNNNNNNAAGGAGGNNNNNNATGGTGAGCAAGGGCGAGG	Fw primer for generating RBS library design 2 in pATM2
oligoATM54	NNNNNNNNNNNNNNNNNAGGAGGNNNNNNATGGTGAGCAAGGGCGAGG	Fw primer for generating RBS library design 3 in pATM2
oligoATM55	NNNNNNNNNNNNNNNNNNGGAGGNNNNNNATGGTGAGCAAGGGCGAGG	Fw primer for generating RBS library design 4 in pATM2
oligoATM56	NNNNNNNNNNNNNNNNNNNGAGGNNNNNNATGGTGAGCAAGGGCGAGG	Fw primer for generating RBS library design 5 in pATM2
oligoATM57	NNNNNNNNNNNNNNNNNNNNAGGNNNNNNATGGTGAGCAAGGGCGAGG	Fw primer for generating RBS library design 6 in pATM2
oligoATM58	NNNNNNNNNNNNNNNNNNNNNGGNNNNNNATGGTGAGCAAGGGCGAGG	Fw primer for generating RBS library design 7 in pATM2
oligoATM59	NNNNNNNNNNNNNNNNNNNNNNGNNNNNNATGGTGAGCAAGGGCGAGG	Fw primer for generating RBS library design 8 in pATM2
oligoATM60	NNNNNNNNNNNNNNNNNNNNNNNNNNNNNATGGTGAGCAAGGGCGAGG	Fw primer for generating RBS library design 9 in pATM2
oligoGT234	GGGGAAACGCCTGGTATCT	pStA0 Fw sequencing primer

oligoGT235	AGCAAAAACAGGAAGGCAAA	pStA0 Rv sequencing primer
oligoGT339	GTTGAGGACCCGGCTAGG	pStA1 Fw sequencing primer
oligoGT340	TGTGACGGAAGATCACTTCG	pStA1 Rv sequencing primer
oligoGT373	TACTGGGTCTCTCTCCGGCATTGTCTTCcat aactgcagtctcctttaaatcggtttc	Fw primer for constructing pGT270
oligoGT374	/5Phos/ctccaggatccaaagccacg	Rv primer for constructing pGT270
oligoGT375	GAAGACAATGCCGGAGAGAGACCCAGTACCA GTAGGGCAGTGAGCGCAAC	Fw primer for constructing pGT270
oligoGT376	GAAGACTTTAGTAGTATGAGACCGGAAAGGA AACTATGCGGCATCAGAGC	Rv primer for constructing pGT270
oligoGT436	TATGTCAGTAAAAAAAAAGGAGGTTCGTTAT GTGAAGAGCGTAAGACCTCTAGGGCGGCG	Fw primer for cloning RBSc4 into pStA0 as a composite part
oligoGT437	TACGAAAGATTTACGAAAGGAGGTTGGTTAT GTGAAGAGCGTAAGACCTCTAGGGCGGCG	Fw primer for cloning RBSc21 into pStA0 as a composite part
oligoGT438	AGTGTGTTCCAAGAGAAAGGAGGTATCTGAT GTGAAGAGCGTAAGACCTCTAGGGCGGCG	Fw primer for cloning RBSc48 into pStA0 as a composite part
oligoGT439	TAGGTATATGCAATCAAAGGAGGTATATGAT GTGAAGAGCGTAAGACCTCTAGGGCGGCG	Fw primer for cloning RBSc110 into pStA0 as a composite part
oligoGT440	TGTGTAGGTCCCACCAAAGGAGGTCTGTAAT GTGAAGAGCGTAAGACCTCTAGGGCGGCG	Fw primer for cloning RBSc123 into pStA0 as a composite part
oligoGT441	GGCGCGAGTCAACGTTCCTGAGGAGTGGCAT GTGAAGAGCGTAAGACCTCTAGGGCGGCG	Fw primer for cloning RBSc249 into pStA0 as a composite part
oligoGT442	AGGTATATTATACTGTTGGGGCAGTTACTTG TCAAGGTCATCTGGCTGTGAAGAGCCACACT GGATTCTCACCAATAAAAAACGC	Rv primer for cloning SPLc3 into pStA0 as a composite part
oligoGT443	AACTAAATTATACTGATATTTAGTTAAATTG TCAATACGAGCCGCCTGTGAAGAGCCACACT GGATTCTCACCAATAAAAAACGC	Rv primer for cloning SPLc19 into pStA0 as a composite part
oligoGT444	ACCTTAATTATATCAGAGCGCAGAGTGCTTG TCAATTTTTCGTGGCTGTGAAGAGCCACACT GGATTCTCACCAATAAAAAACGC	Rv primer for cloning SPLc25 into pStA0 as a composite part

oligoGT445	ACGTTTATTATATAGTTAGCGGGCCGCTGTGTCAAAGGTGGCGAGCTGTGAAGAGCCACACTGGATTCTCACCAATAAAAAACGC	Rv primer for cloning SPLc45 into pStA0 as a composite part
oligoGT446	TATTCCATTATATCTCGGTAACTCCTGGCTGTCAATAGACGTCACCTGTGAAGAGCCACACTGGATTCTCACCAATAAAAAACGC	Rv primer for cloning SPLc47 into pStA0 as a composite part
oligoGT578	CGGCGGGTTTTTTTATAGCTAAAAGGACGAAGAGCGACCTCTAGGGCGGCGG	Fw primer for cloning ECK120015170 into pStA0
oligoGT579	AAGCGGGTTTTTTCGAAAATTGTTTATGAAGAGCCACACTGGATTCTCACCAATAAAAAACGC	Rv primer for cloning ECK120015170 into pStA0
oligoGT583	/5Phos/CGAATCATTATAGGGTTGTGTTCTCTGCGTGTCAAAGCTTAGGGGCTGTGAAGAGCCACACTGGATTCTCACCAATAAAAAACGC	Rv primer for cloning SPLc17 into pStA0 as a composite part
oligoGT603	CCATAAAGCCAACCACACCT	Rv primer to screen for presence of lycopene pathway in *ssl0410* locus
oligoGT605	TTGATCGAACGGGGTGTTAT	Fw primer to screen for presence of lycopene pathway in *ssl0410* locus
oligoGT653	GATCTGGCATGGGTGGAG	Rv primer to screen for presence of lycopene pathway in *ssl0410* locus
oligoGT654	ACCCTGAATGATGCTCTCCA	Fw primer to screen for presence of lycopene pathway in *ssl0410* locus
oligoAH34	CAGGAAATATTGCTTTGCAGG	Fw sequencing primer for pATM2 and all derivative plasmids
oligoAH35	ACACCCCTTGTATTACTGTTTATGT	Rv sequencing primer for pATM2 and all derivative plasmids
oligoAH48	ACTTTGGTTGCTACCTCGCT	Fw primer to screen for integration into *ssl0410* locus
oligoAH49	AGGGAAAATGCCAAAACGCA	Rv primer to screen for integration into *ssl0410* locus

Table S4. Table of plasmids used in this study.

Plasmid	Relevant properties or Description	Source
pATM2	pSHUTTLE2 derivative with promoter P*sll1120* and EYFP reporter	[2]
pATM10	pSHUTTLE2 derivative with promoter P*psbA2* and EYFP reporter	This study
pStA0	Start-Stop Assembly empty storage vector	[1]
pStA1AB	Start-Stop Assembly Level 1 vector (A and B fusion sites)	[1]
pStA1BC	Start-Stop Assembly Level 1 vector (B and C fusion sites)	[1]
pStA1CD	Start-Stop Assembly Level 1 vector (C and D fusion sites)	[1]
pStA1DZ	Start-Stop Assembly Level 1 vector (D and Z fusion sites)	[1]
pStA1DZ	Start-Stop Assembly Level 1 vector (D and Z fusion sites)	[1]
pStA212	Start-Stop Assembly Level 2 vector (1 and 2 fusion sites)	[1]
pGT270	pSHUTTLE2 derivative with pStA212 Start-Stop Assembly cassette	This study
pGT287	pStA0::SPLc3-RBSc4 (composite promoter-RBS part)	This study
pGT288	pStA0::SPLc19-RBSc4 (composite promoter-RBS part)	This study
pGT289	pStA0::SPLc25-RBSc4 (composite promoter-RBS part)	This study
pGT290	pStA0::SPLc45-RBSc4 (composite promoter-RBS part)	This study
pGT293	pStA0::SPLc3-RBSc21 (composite promoter-RBS part)	This study
pGT294	pStA0::SPLc19-RBS21 (composite promoter-RBS part)	This study
pGT295	pStA0::SPLc25-RBSc21 (composite promoter-RBS part)	This study
pGT296	pStA0::SPLc45-RBSc21 (composite promoter-RBS part)	This study
pGT297	pStA0::SPLc47-RBS21 (composite promoter-RBS part)	This study
pGT300	pStA0::SPLc19-RBS48 (composite promoter-RBS part)	This study
pGT301	pStA0::SPLc25-RBSc48 (composite promoter-RBS part)	This study
pGT302	pStA0::SPLc45-RBSc48 (composite promoter-RBS part)	This study
pGT303	pStA0::SPLc47-RBSc48 (composite promoter-RBS part)	This study
pGT305	pStA0::SPLc3-RBSc110 (composite promoter-RBS part)	This study
pGT306	pStA0::SPLc19-RBSc110 (composite promoter-RBS part)	This study
pGT307	pStA0::SPLc25-RBSc110 (composite promoter-RBS part)	This study

pGT308	pStA0::SPLc45-RBSc110 (composite promoter-RBS part)	This study
pGT309	pStA0::SPLc47-RBSc110 (composite promoter-RBS part)	This study
pGT311	pStA0::SPLc3-RBSc123 (composite promoter-RBS part)	This study
pGT312	pStA0::SPLc19-RBSc123 (composite promoter-RBS part)	This study
pGT313	pStA0::SPLc25-RBSc123 (composite promoter-RBS part)	This study
pGT314	pStA0::SPLc45-RBSc123 (composite promoter-RBS part)	This study
pGT315	pStA0::SPLc47-RBSc123 (composite promoter-RBS part)	This study
pGT317	pStA0::SPLc3-RBSc249 (composite promoter-RBS part)	This study
pGT318	pStA0::SPLc19-RBSc249 (composite promoter-RBS part)	This study
pGT319	pStA0::SPLc25-RBsc249 (composite promoter-RBS part)	This study
pGT320	pStA0::SPLc45-RBsc249 (composite promoter-RBS part)	This study
pGT321	pStA0::SPLc47-RBSc249 (composite promoter-RBS part)	This study
pGT338	pStA0::L3S2P21	[1]
pGT339	pStA0:: ECK120033737	[1]
pGT340	pStA0:: ECK120029600	[1]
pGT356	pStA0::*crtI*	[1]
pGT357	pStA0::*crtB*	[1]
pGT358	pStA0::*crtE*	[1]
pGT359	pStA0::*dxs*	[1]
pGT424	pStA0::ECK120015170	This study
pGT425	pStA0::SPLc17-RBSc4 (composite promoter-RBS part)	This study
pGT426	pStA0::SPLc17-RBSc21 (composite promoter-RBS part)	This study
pGT427	pStA0::SPLc17-RBSc48 (composite promoter-RBS part)	This study
pGT428	pStA0::SPLc17-RBSc110 (composite promoter-RBS part)	This study
pGT429	pStA0::SPLc17-RBSc123 (composite promoter-RBS part)	This study
pGT430	pStA0::SPLc17-RBSc249 (composite promoter-RBS part)	This study
pGT432	High expression design for lycopene pathway	This study
pGT433	Medium expression design for lycopene pathway	This study
pGT434	Low burden design for lycopene pathway	This study

pGT435	High burden design for lycopene pathway	This study
pGT437	Lycopene pathway clone randomly chosen from pGT547	This study
pGT438	Lycopene pathway clone randomly chosen from pGT547	This study
pGT439	Lycopene pathway clone randomly chosen from pGT547	This study
pGT440	Lycopene pathway clone randomly chosen from pGT547	This study
pGT441	Lycopene pathway clone randomly chosen from pGT547	This study
pGT442	Lycopene pathway clone randomly chosen from pGT547	This study
pGT443	Lycopene pathway clone randomly chosen from pGT547	This study
pGT444	Lycopene pathway clone randomly chosen from pGT547	This study
pGT445	Lycopene pathway clone randomly chosen from pGT547	This study
pGT446	Lycopene pathway clone randomly chosen from pGT547	This study
pGT447	Lycopene pathway clone randomly chosen from pGT547	This study
pGT448	Lycopene pathway clone randomly chosen from pGT547	This study
pGT449	Lycopene pathway clone randomly chosen from pGT547	This study
pGT450	Lycopene pathway clone randomly chosen from pGT547	This study
pGT451	Lycopene pathway clone randomly chosen from pGT547	This study
pGT452	Lycopene pathway clone randomly chosen from pGT547	This study
pGT453	Lycopene pathway clone randomly chosen from pGT547	This study
pGT454	Lycopene pathway clone randomly chosen from pGT547	This study
pGT455	Lycopene pathway clone randomly chosen from pGT547	This study
pGT456	Lycopene pathway clone randomly chosen from pGT547	This study
pGT457	Lycopene pathway clone from pGT547 with prescreen	This study
pGT458	Lycopene pathway clone from pGT547 with prescreen	This study
pGT459	Lycopene pathway clone from pGT547 with prescreen	This study
pGT460	Lycopene pathway clone from pGT547 with prescreen	This study
pGT461	Lycopene pathway clone from pGT547 with prescreen	This study
pGT462	Lycopene pathway clone from pGT547 with prescreen	This study
pGT463	Lycopene pathway clone from pGT547 with prescreen	This study
pGT464	Lycopene pathway clone from pGT547 with prescreen	This study

pGT465	Lycopene pathway clone from pGT547 with prescreen	This study
pGT466	Lycopene pathway clone from pGT547 with prescreen	This study
pGT467	Lycopene pathway clone from pGT547 with prescreen	This study
pGT468	Lycopene pathway clone from pGT547 with prescreen	This study
pGT469	Lycopene pathway clone from pGT547 with prescreen	This study
pGT470	Lycopene pathway clone from pGT547 with prescreen	This study
pGT471	Lycopene pathway clone from pGT547 with prescreen	This study
pGT472	Lycopene pathway clone from pGT547 with prescreen	This study
pGT473	Lycopene pathway clone from pGT547 with prescreen	This study
pGT474	Lycopene pathway clone from pGT547 with prescreen	This study
pGT475	Lycopene pathway clone from pGT547 with prescreen	This study
pGT476	Lycopene pathway clone from pGT547 with prescreen	This study
pGT517	Lycopene pathway clone from pGT547 with prescreen	This study
pGT518	Lycopene pathway clone from pGT547 with prescreen	This study
pGT519	Lycopene pathway clone from pGT547 with prescreen	This study
pGT520	Lycopene pathway clone from pGT547 with prescreen	This study
pGT547	Lycopene pathway library	This study

Table S5. List of synthetic DNA sequences used in the study

Synthetic DNA sequence name	Sequence (5'-3')
crtI recoded (1647 bp)	atgacatcagctctcccccgcccggcaccaagtccgtacgcacgccgtaaaac<br>ggcgttggttattggcgcaggttttggtgggttggccctgggcattcgtctac<br>agtcgttaggttttgatacaacaattttggaacgtctggatggtcctggtggt<br>cgcgcgtatcaaaaacgtaccccagatggctatgtctttgacatgggtccgac<br>tgtgctgacggtgccgcattttatcgaagaactgtttgcgcttgaacgtgatc<br>gtgccggcctggatgcccccgattatcctcctgaagtgttgtcgggcgagcgc<br>gttaaggaaggcgtttctggtggcccgcatacgagccggtatgtcaccttagt<br>gccgattctgcccttttaccgcattgtttttcacgatggcacgtattttgatt<br>atgatggcgaccctgaaagtactcggcggcagattgctgaattggcccctggc<br>gacttagccgggtatgaacgctttcatgccgatgccgaggccatctttcgtcg<br>gggcttcctggaactgggctacacgcactttggtgacgtcccgacgatgctgc<br>gggttgtacctgatctgctcaaactggacgccgttcggaccctgttctccttt<br>acgagtaagtactttcagagcgacaaactgcgccaagtgttctcttttgaaac<br>ccttttggtgggtgggaatcctctgagtgtgccggcgatctatgcaatgattc<br>acttcgttgaaaagacttgggggatccactatgccatgggcggcacaggcgca<br>ctggtgcgcggcctagtccaaaaatttgaggagctgggtggcgccattcgtta<br>tggcgccggcgtcgatgaagtactggtggatggcaatctgcctggtaaacgca<br>cagcgcggggtgtgcgcctggaaagcggcgaagaactgcgcgccgacctggtg<br>gcgtccaatggcgattgggctaacacgtatctgaaacgcgtccggccatcggc<br>acgtctggtcaactctgatttacgcgtgaaagccgcatctgaaagtatgagcc<br>tgctcgtggtttatttcgggtttcgcggcggtgatgacctgcccctcaaacat<br>cataatattttattaggcccacgctacgaggctctgctgagcgaaatctttgg<br>cacaaaacggttgggcgaagattttagccagtacctgcacgtcccaacgctca<br>ccgatccggctctggcacccgcgggtcatcatgcggcctatacacttgtcccg<br>gtgccgcataatggctcgggtattgattgggacgtggaaggtccaaagcttgc<br>cgaagcagccctggcagatatcgagcgccgcggtttgattccgggcctccgtg<br>aacggctcacacattttgaatttattacgccagattatttcgcaggcactctc<br>gattcctatctggggaacgcgtttggtccggagccgcgtctggtccagtcggc<br>atttttccgcccgcacaaccgcagcgaggatctccacaactttacttagtcg<br>gggcgggcgcgcagccaggcgcaggcacaccgagcgttatgatgtccgcgaaa<br>atgacagcgcgcctaatcgctgaagatttcggtatccatgctgatatccggcg<br>ctaa

crtB recoded (978 bp)	atgcgtagtcgcgctggtctgagcttacggttacccacgcgtaccttgaccgt<br>gaccgattactcccccgccctgccctgcaccgaactgcgccgtcctccactgg<br>ctcaggcggttcgctactgtcgggatttgacccgccagcactcaaagaccttc<br>tatctgggttcacagctcttttcgcctccggaacgcgcggcagtttgggcagt<br>gtatgcggcgtgccgcgcgggcgatgacatcgtcgatgaagccggcaacggcg<br>accgcgaacgcgaattgcgggaatggcgcagccgtattgatgccgcgtttgct<br>ggccaaccagcggatgatcccatctcaaccgcgctggcctgggcggcaggtcg<br>gtacgccatcccgcactcagctttcgcggaactgcatgaaggcctcaacatgg<br>atttacgcggtcatgaataccgtgatatggatgacttgttactgtattgccgc<br>cgtgtggcaggtgtggttggctttatggtggcaccgatttctggctaccgtgg<br>gggggctgctaccctgaatgatgctctccaactagggcaggcgatgcaactga<br>cgaatattctgcgcgatgtcggtgaagatctgacccgcggccgcgtatacctg<br>ccacagtctctgcttgatgaatatggcctgtctcgcgccgcgttagagcgctg<br>gggtcagggtgagcccctgtcaccggcctaccgtgctctcatgactcatcttg<br>gcggccttgcacgtgaatggtatgcagcaggtcgtgctggtattcctcaactt<br>gatggacgcggtcctctcgccgttctgactgccgcccgtgcgtatgagggtat<br>tctggacgatttggaacgggccggctacgacaacttcggtcggcgcgcgtacg<br>tgtcaggtcgtcgtaaacttctgatgttaccgcaggcctggtgggaactgcgt<br>agtctgggcgctgtccacggctaa
crtE recoded (990 bp)	atgcgcccggaattactcgcacgcgtgttaagcctgttaccggaaacctccgc<br>gacgccggaattggcacgcttttacgcgctcctgcgcgactatcctcaacgtg<br>gtggcaagggcattcggtcagaattactgcttgcctctgctcgtgcgcacggc<br>ctgtccgagtcagataccggttgggagtcagcattatggctggcggcagcctt<br>agaactgtttcagaactgggtgctggtgcacgatgatattgaagatgattcgg<br>aagaacgccgtggtcgtccggccctgcaccacttgtgtggtatgccggtcgct<br>cttaacgtgggggacgcgctgcacgcttacatgtgggctgctgttgggaaagc<br>caatgttccgggagcgtttgaagagtttctgcagatggtgtaccgcacggcgg<br>aaggccagcatctggatctggcatgggtggagggtcgtgaatggggcctgcgt<br>cccgcggattatctccagatggttggcctgaaaccgcacactacacggttat<br>cgtgccgttacgtctgggggccctggcggcaggcatggcaccgcaggacgcgt<br>tcaccccagcgggtctggcgctgggtaccgcgtttcagattcgtgacgatgtc<br>ctcaatctggcaggtgatccggtgaagtatggtaaagaaattggtggcgatct<br>gttggaaggtaaacgtactctgattgtcctggactggttgactacggcgccgg<br>atgatcgcaaagccatcttcctggaccagatgcgtcggcaccgcgcagataaa<br>gaccctgcggtgatcgatgaaattcaccgctggctgcttgaaagcggctctgt<br>ggaagcggcgcaggactacgcgcaggcacaagccgcggaaggtctggacttgc<br>ttgaaaaagcattggcagacgcgccggatgcccaggccgccgctgccttactc<br>gcttctgttcgggaactggccacccgcgaaaaataa
dxs recoded (1863 bp)	atgtcttttgatattgcgaaatatccaaccctggccctagttgactcgactca<br>ggaattacgcctgctgccgaaagagagccttccaaagctgtgcgatgagttac<br>gccgctacctcctggattctgttagtcgtagctccggccacttcgcatcggga<br>ctaggtaccgtcgaactgacggttgcactgcattatgtgtataacaccccgtt<br>cgatcaattaatttgggacgtcggtcatcaagcatatccgcataaaattctga

ccggtcgtcgcgacaagatcggcacaattcgtcaaaaaggtggattgcatcct
ttcccgtggcgcggcgagtccgaatacgatgtgttgagcgttggccattcgtc
aacttctatcagcgctggtattggtattgcggtcgccgcggagaaagaaggca
aaaatcggcgtacggtttgcgtgatcggggatggtgcaatcaccgcaggcatg
gcattcgaagcgatgaaccatgcgggggacattcgtccggatatgctagtgat
tctgaacgataacgaaatgagtatttccgagaacgtggggctcttaataacc
acttagcgcagctgctgagcggtaaactttattctagcctgcgcgaaggcggt
aagaaagtgttctcaggcgtccctccgattaaagagctccttaagcgtactga
ggaacacattaagggtatggtggttccaggcaccctgttcgaagaactgggtt
ttaattacattggtcctgtggacggccatgacgtgttaggcttaattaccacg
ttaaaaaacatgcgcgatctgaaaggacctcagtttctccacatcatgaccaa
gaaaggtcgcggctatgaaccggccgagaaagatccgattaccttccacgcag
ttccgaaattcgacccatcctccggctgtctgccgaaaagcagcggaggcctg
ccgagctattccaaaatctttggtgactggctgtgcgaaactgcggcaaaaga
taacaaactgatggccatcaccccggcaatgcgcgaaggttcgggaatggtgg
aattctctcgcaagttcccagaccgttattttgacgttgctatcgccgagcaa
catgcagtaacctttgcggctggtctggcaattggtggttataagccaattgt
ggccatctattcaacgttcttacaacgtgcatatgaccaggtgctacatgatg
tggccattcagaaactgccggtcttatttgccatcgatcgtgcggggatcgtt
ggggcagatggtcagacgcatcagggcgccttcgacctaagttatctgcgctg
tatcccagaaatggtgattatgacccctagcgatgagaatgaatgtcgtcaga
tgttatacaccggctatcattacaacgacggaccatccgctgtgcgctacccg
cgcggaaacgcggtgggcgttgaactgaccccgttagaaaaattaccgatcgg
aaaagggattgttaaacgtcgcggagaaaaactcgctattctgaacttcggca
cacttatgcccgaagcggctaaagtagcggaaagcctaaacgcaaccttggtg
gatatgcgcttcgtaaagccactggacgaagccctaatcttagagatggccgc
gtcacatgaggcgttagttacggtagaggaaaacgctattatgggcggagcgg
gtagtggtgtgaacgaagttctgatggctcaccgcaaaccggtgccggtactg
aacatcggcctcccggatttttcattccccaaggtactcaagaggaaatgcg
cgccgagctcggcttagatgctgcaggaatggaagccaaaattaaggcctggc
ttgcataa

Table S6. Promoters and RBSs stored in pStA0. Fw primer and Rv primer signify the oligonucleotides used for the inverse PCR carried out to generate the given construct.

Plasmid ID	Promoter	RBS	Fw primer	Rv primer
pGT287	SPLc3	RBSc4	oligoGT436	oligoGT442
pGT288	SPLc19	RBSc4	oligoGT436	oligoGT443
pGT289	SPLc25	RBSc4	oligoGT436	oligoGT444
pGT290	SPLc45	RBSc4	oligoGT436	oligoGT445
pGT293	SPLc3	RBSc21	oligoGT437	oligoGT442
pGT294	SPLc19	RBSc21	oligoGT437	oligoGT443
pGT295	SPLc25	RBSc21	oligoGT437	oligoGT444
pGT296	SPLc45	RBSc21	oligoGT437	oligoGT445
pGT297	SPLc47	RBSc21	oligoGT437	oligoGT446
pGT300	SPLc19	RBSc48	oligoGT438	oligoGT443
pGT301	SPLc25	RBSc48	oligoGT438	oligoGT444
pGT302	SPLc45	RBSc48	oligoGT438	oligoGT445
pGT303	SPLc47	RBSc48	oligoGT438	oligoGT446
pGT305	SPLc3	RBSc110	oligoGT439	oligoGT442
pGT306	SPLc19	RBSc110	oligoGT439	oligoGT443
pGT307	SPLc25	RBSc110	oligoGT439	oligoGT444
pGT308	SPLc45	RBSc110	oligoGT439	oligoGT445
pGT309	SPLc47	RBSc110	oligoGT439	oligoGT446
pGT311	SPLc3	RBSc123	oligoGT440	oligoGT442
pGT312	SPLc19	RBSc123	oligoGT440	oligoGT443
pGT313	SPLc25	RBSc123	oligoGT440	oligoGT444
pGT314	SPLc45	RBSc123	oligoGT440	oligoGT445
pGT315	SPLc47	RBSc123	oligoGT440	oligoGT446
pGT317	SPLc3	RBSc249	oligoGT441	oligoGT442
pGT318	SPLc19	RBSc249	oligoGT441	oligoGT443
pGT319	SPLc25	RBSc249	oligoGT441	oligoGT444
pGT320	SPLc45	RBSc249	oligoGT441	oligoGT445

pGT321	SPLc47	RBSc249	oligoGT441	oligoGT446
pGT425	SPLc17	RBSc4	oligoGT436	oligoGT583
pGT426	SPLc17	RBSc21	oligoGT437	oligoGT583
pGT427	SPLc17	RBSc48	oligoGT438	oligoGT583
pGT428	SPLc17	RBSc110	oligoGT439	oligoGT583
pGT429	SPLc17	RBSc123	oligoGT440	oligoGT583
pGT430	SPLc17	RBSc249	oligoGT441	oligoGT583

Supplementary References

1. Taylor, G. M., Mordaka, P. M. & Heap, J. T. Start-Stop Assembly: a functionally scarless DNA assembly system optimized for metabolic engineering. *Nucleic Acids Res.* **47**, e17 (2018).
2. Kelly, C. L., Taylor, G. M., Hitchcock, A., Torres-Méndez, A. & Heap, J. T. A Rhamnose-Inducible System for Precise and Temporal Control of Gene Expression in Cyanobacteria. *ACS Synth. Biol.* (2018) doi:10.1021/acssynbio.7b00435.
3. Salis, H. M., Mirsky, E. A. & Voigt, C. A. Automated design of synthetic ribosome binding sites to control protein expression. *Nat. Biotechnol.* **27**, 946–950 (2009).
